# Gamma-Glutamyltransferase and Risk of Acute Coronary Syndrome in Young Chinese Patients: A Case-Control Study

**DOI:** 10.1155/2018/2429160

**Published:** 2018-09-02

**Authors:** Yuli Huang, Jianjin Luo, Xinyue Liu, Yu Wu, You Yang, Wensheng Li, Weibiao Lv, Yunzhao Hu

**Affiliations:** ^1^Department of Cardiology, Shunde Hospital, Southern Medical University (the First People's Hospital of Shunde), Foshan, China; ^2^Department of Internal Medicine, Zhaoqing Medical College, Guangdong, China; ^3^Department of Clinical Laboratory, Shunde Hospital, Southern Medical University (the First People's Hospital of Shunde), Foshan, China

## Abstract

**Background:**

Serum gamma-glutamyltransferase (GGT) is a biomarker of hepatic disease. Recent studies have shown that GGT may also associate with the risk of coronary artery disease. However, the underlying mechanisms of this association are still unclear.

**Methods:**

This study included 216 young patients with acute coronary syndrome (aged ≤55years) and 227 age-matched controls with normal findings by coronary angiography or coronary computed tomography angiography. We use standard colorimetric techniques and sandwich enzyme-linked immunosorbent assay to measure the levels of GGT and oxidized low-density lipoprotein (ox-LDL), respectively. Traditional risk factors of coronary artery disease, including smoking, diabetes mellitus, hypertension, dyslipidemia, and obesity/overweight, were evaluated according to the current guidelines.

**Results:**

The levels of GGT were significantly correlated with body mass index and levels of triglyceride, fasting plasma glucose, aspartate aminotransferase, and ox-LDL (all *P* < 0.05). Multivariate logistic regression analysis showed that GGT was significantly associated with the risk of acute coronary syndrome in young Chinese patients (OR = 1.53, 95% CI = 1.09–2.15) after adjusting for traditional risk factors, including sex, age, quantity of smoking, hypertension, diabetes, body mass index, dyslipidemia, and high-sensitivity C-reactive protein. However, this association was significantly attenuated (OR = 1.20, 95% CI = 0.91–1.58) after further adjusting for the levels of ox-LDL.

**Conclusions:**

GGT was associated with the risk of ACS in relatively young patients. The link between GGT and the risk of ACS may be dependent on ox-LDL levels, indicating that the prooxidant action is an important pathway for GGT in the development of cardiovascular disease.

## 1. Introduction

Coronary artery disease (CAD) is a major cause of death worldwide [1]. It has been conventionally associated with older age. However, patients with CAD attack at a younger age have been greatly increasing during the past decades [2, 3]. There are many differences, both in the clinical characteristics and in the risk factors between young and older patients with CAD. Young patients often develop acute coronary syndrome (ACS) but not stable CAD [4]. Furthermore, young patients with CAD are less associated with the conventional risk factors, such as hypertension, dyslipidemia, and diabetes mellitus [5]. It had been reported that none or only one conventional CAD risk factor can be found in 36% of young patients with ACS. According to the traditional risk scoring system, these patients would be defined as with low risk of CAD [6]. In order to better prevent premature CAD, novel risk factors and disease markers should be evaluated.

Gamma-glutamyltransferase (GGT), a widely used biomarker for excessive alcohol consumption and fatty liver disease, is reportedly associated with an increased risk of cardiovascular disease [7–9]. Further analysis has shown that GGT may more accurately predict cardiovascular disease in young than in elderly patients [8, 10, 11]. Our prior study showed that the oxidized low-density lipoprotein (ox-LDL) level is an important risk factor for CAD in young patients [12]. Interestingly, GGT was detected within atherosclerotic plaques of coronary arteries, where it colocalizes with ox-LDL [13]. However, the correlation between GGT and ox-LDL levels has not been evaluated in young patients with ACS, and whether the effect of GGT on the incidence of ACS is mediated by or independent of ox-LDL is unclear.

Therefore, we evaluated the association between GGT and the risk of ACS in young Chinese patients, as well as the correlation between GGT and ox-LDL, in this study.

## 2. Materials and Methods

### 2.1. Participants

All the participants in the ACS and control groups were inpatients from September 2012 to September 2015 in the Shunde Hospital, Southern Medical University, China. Young patients (≤55 years of age) with a first attack of myocardial ischemia within 2 months and at least one major coronary artery with lumen diameter ≥ 50% stenosis would be diagnosed as ACS [14]. The coronary artery stenosis was quantified by coronary angiography (CAG), using the Judkins technique with the Allura Xper FD20 (Philips, Amsterdam, Netherlands) by two independent interventional cardiologists.

The control group contained age-matched individuals with negative findings by ultrafast coronary computed tomography angiography (CCTA) or normal findings with CAG. They were hospitalized for chest pain with ischemic findings (e.g., ST deviation on electrocardiography, positive treadmill test results, regional dyskinesia on ultrasonic cardiography, and ischemic signs by single-photon emission computed tomography) or difficult-to-make differential diagnosis [14].

The exclusion criteria were as follows: (1) suspected acute myocardial infarction (defined by chest pain and elevated troponin) but without obstructive coronary stenosis (these patients had diverse pathophysiological mechanisms different from those involving obstructive CAD) [15]; (2) uncontrolled infectious disease, autoimmune disease, severe renal dysfunction (serum creatinine of ≥265 *μ*mol/L), history of hepatitis or positive detection of serum hepatitis B virus antigen, psychiatric disorders, malignancy, pregnancy, or hormone replacement therapy after menopause; and (3) a history of alcohol abuse (defined as alcohol consumption of ≥100 g/day).

The study complied with the Declaration of Helsinki and was approved by the Ethics Committee of Shunde Hospital, Southern Medical University, China. Written informed consent was obtained from all participants.

### 2.2. Laboratory Measurements

Venous blood samples were collected after overnight fasting at the second day when participants were hospitalized for chest pain. We use an AU2700 Automatic Biochemical Analyzer (Olympus, Tokyo, Japan) to measure the levels of triglyceride, fasting plasma glucose (FPG), serum creatinine (Scr), total cholesterol (TC), low-density lipoprotein cholesterol (LDL-C) and high-density lipoprotein cholesterol (HDL-C), and total bilirubin (TBIL) and direct bilirubin (DBIL). Levels of indirect bilirubin (IBIL) were calculated from levels of TBIL and DBIL. Serum GGT, alanine aminotransferase (ALT), and aspartate aminotransferase (AST) were measured using an enzymatic colorimetric test with a Hitachi 7600 analyzer (Hitachi, Tokyo, Japan). High-sensitivity C-reactive protein (hs-CRP) was measured by the high-sensitivity nephelometric method. Plasma samples were centrifugated at 1500 × *g* for 10 min and stored at −80°C for future measurements of ox-LDL levels by sandwich enzyme-linked immunosorbent assay (Mercodia, Uppsala, Sweden) [12]. Coefficients of variation for intra-assay and interassay for GGT were respectively 3.4% and 7.8% and for ox-LDL were 2.1% and 8.4%.

### 2.3. Definition of Conventional Risk Factors for CAD

Conventional risk factors of CAD evaluated in this study were as follows. (1) Family history of premature cardiovascular disease was defined as positive if cardiovascular disease is existing in a first-degree male relative aged <55 years or female relative aged <65 years. (2) Hypertension was defined according to the Joint National Committee on Prevention, Detection, Evaluation, and Treatment of High Blood Pressure VII guidelines [16]. (3) Participants were defined as current smokers (if they reported smoking regularly during the past 1 year preceding the enrollment), past smokers (if they had stopped smoking at least 1 year), and nonsmokers. For participants who reported past or current cigarette smoking, information on the number of cigarettes smoked was also collected and quantified as pack years from the number of packs per day multiplied by number of years smoked; one pack contains 20 cigarettes [12]. (4) Diabetes mellitus was diagnosed according to the current American Diabetes Association criteria [17]. (5) According to the Guidelines for Chinese, participants with a history of receiving antidyslipidemia agents or with a level of TC ≥ 200 mg/dL, HDL-C < 40 mg/dL, LDL-C ≥ 130 mg/dL, and/or triglyceride ≥ 150 mg/dL were diagnosed with dyslipidemia [18]. (6) Based on the Chinese criteria, participants with a body mass index of 24.0 to 27.9 kg/m^2^ would be defined as overweight and those of ≥28 kg/m^2^ as obese [19]. (7) The estimated glomerular filtration rate (eGFR) was calculated based on the modified Modification of Diet in Renal Disease equation adapted for Chinese [20]. (8) The absolute 10-year cardiovascular risk scores were evaluated based on the Framingham Risk Score system [21].

### 2.4. Statistical Analysis

We used SPSS Statistics 22.0 for Windows (IBM Corp., Armonk, NY, USA) to perform all statistical analyses. The chi-square or Fisher's exact test was used to compare categorical variables, which were presented as percentages. After testing for normality, continuous variables were expressed as median (interquartile range) or mean (standard deviation) and compared using the Mann–Whitney *U* test or Student's *t*-test. Skewed variables were logarithmically transformed (GGT, AST, ALT, and hs-CRP) and analyzed as a continuous variable.

We used the Spearman rho and Pearson tests to evaluate the correlations between variables. Multiple logistic regression analysis was used to evaluate the risk factors for ACS. A family history of CAD, pack years of smoking, age, hypertension, diabetes mellitus, body mass index, triglyceride, HDL-C, LDL-C, eGFR, and hs-CRP were set as independent variables in the regression model. Odds ratios (ORs) and 95% confidence intervals (CIs) were reported. “Statistically significant” was defined as the *P* value of <0.05. Multicollinearity (the strong correlations of independent variables) was tested according to collinearity diagnostic statistics. In logistic regression models, a tolerance of <0.4 or variance inflation factor of >2.5 suggests the presence of multicollinearity [22].

## 3. Results

### 3.1. Patients' Clinical Characteristics

261 young patients (≤55 years old) with ACS and 266 age-matched controls were screened. We excluded 45 patients (uncontrolled infectious disease, *n* = 14; history of hepatitis B, *n* = 13; alcoholic, *n* = 10; and severe renal dysfunction, *n* = 8) and 39 controls (uncontrolled infectious disease, *n* = 6; history of hepatitis B, *n* = 15; alcoholic, *n* = 8; severe anxiety, *n* = 7; and severe renal dysfunction, *n* = 3). Finally, 216 young patients diagnosed with ACS and 227 age-matched controls were analyzed in this study. Among all of the patients with ACS, 65 had non-ST-segment elevation myocardial infarction, 91 had ST-segment elevation myocardial infarction, and 60 had unstable angina.  Table 1 shows all participants' clinical characteristics. Compared with controls, patients diagnosed with ACS were characterized by a greater percentage of males, dyslipidemia, smoking, and higher levels of triglyceride, AST, ALT, GGT, hs-CRP, and ox-LDL (all *P* < 0.05). No other differences were observed in CAD risk factors between the two groups.

### 3.2. Correlations between GGT and Other Risk Factors in Young Patients with ACS

Table 2 presents the correlations between GGT and other risk factors in young patients. There is a positive correlation between GGT levels and other risk factors, such as fasting plasma glucose (*r* = 0.22, *P* = 0.02), body mass index (*r* = 0.18, *P* = 0.04), ox-LDL (*r* = 0.32, *P* < 0.001), AST (*r* = 0.23, *P* = 0.04), and triglycerides (*r* = 0.23, *P* = 0.01), but not with age, quantity of smoking, hs-CRP, eGFR, or systolic blood pressure (all *P* > 0.05).

### 3.3. GGT Level and Risk of ACS in Young Patients

As shown in Table 3, according to multivariate logistic regression analysis, quantity of smoking (OR = 2.79, 95% CI = 1.48–5.30), male sex (OR = 3.45, 95% CI = 1.21–9.87), triglycerides (OR = 1.48, 95% CI = 1.03–2.13), and hs-CRP (OR = 2.36, 95% CI = 1.18–4.72) were independently related with the risk of ACS in young patients. GGT was also associated with the risk of ACS (OR = 1.53, 95% CI = 1.09–2.15) after adjusting for conventional risk factors, including CAD family history, age, sex, quantity of smoking, fasting plasma glucose, body mass index, systolic blood pressure, TC, LDL-C, triglycerides, and hs-CRP. However, when further adjusted for the levels of ox-LDL, the association between GGT and the risk of ACS was insignificant (OR = 1.20, 95% CI = 0.91–1.58). Furthermore, the multicollinearity analysis showed a tolerance factor of <0.4, suggesting that in the multivariate logistic regression models, there is no significant multicollinearity.

## 4. Discussion

The main outcomes of the present study are twofold. First, to our knowledge, this is the first study reporting that GGT is associated with the risk of ACS in young Chinese patients after adjustment for multiple conventional risk factors. Second, the link between GGT and premature CVD may be dependent on ox-LDL, a marker of oxidative stress.

In line with our study, Ding et al. reported that GGT levels were significantly associated with the risk of significant coronary stenosis in middle-aged Chinese after adjustment for conventional risk factors, while no significant associations were found with body mass index, waist circumference, or visceral fat areas [23]. However, circulating ox-LDL levels were not evaluated in that study, and the potential mechanisms were not explored. Circulating GGT originates mainly from the liver and is influenced by genetic and environmental factors [24]. GGT is also closely associated with established cardiovascular risk factors such as DM, insulin resistance, and low-grade inflammation [25]. Such putative mechanisms may be involved in the association between GGT and an elevated risk of cardiovascular disease. In our study, however, after adjusting for multiple conventional cardiovascular risk factors including body mass index, fasting plasma glucose, and hs-CRP, the GGT level was still associated with the risk of ACS. The Framingham Study also showed that serum GGT was positively associated with incident cardiovascular disease even after accounting for CRP [26]. These results indicate that other mechanistic pathways should be considered in the association between GGT and the risk of cardiovascular disease.

GGT is located on the external surface of cellular membranes of various cells and involved in the homeostasis of glutathione, the principal thiol antioxidant in humans [9]. Cleavage of glutathione by GGT in the extracellular space produces cysteinylglycine, which is a powerful reductant of Fe^3+^ and is able to simultaneously generate Fe^2+^ and a free thiyl radical, exerting a local prooxidant action and causing LDL oxidation [27]. Ox-LDL is taken up by the scavenger receptor system, leading to generation of foam cells and development of early lesions. It is a chemoattractant for monocytes and T lymphocytes and inhibits macrophage motility, thereby promoting retention of macrophages in the arterial wall and causing atherosclerosis [12]. In our study, when further adjusting for the ox-LDL levels, the association between GGT and the risk of ACS became insignificant, indicating that the link between GGT and premature cardiovascular disease is, at least in part, dependent on the prooxidant action. These suggestions are supported by a prior study showing that catalytically active GGT colocalized with ox-LDL and CD68^+^ foam cells within atherosclerotic plaques from autoptic studies and surgical endarterectomy [28]. Different from our study, the European Prospective Investigation into Cancer and Nutrition- (EPIC-) Potsdam study showed that cysteinylglycine and ox-LDL only accounted for 2.3% of the relationship between GGT and the risk of myocardial infarction, suggesting that the positive association between GGT activity and the risk of myocardial infarction appears to be independent of ox-LDL levels [25]. The discrepancy between these studies may be caused by different ethnicities and ages. Patients with myocardial infarction in the EPIC-Potsdam study were all European and had a mean age of 56.2 years, while our study included Chinese patients aged <55 years.

GGT measurements are now well standardized, simple, inexpensive and commonly measured as part of routine liver function panels, and do not require a fasting state prior to venous puncture. Although the association between GGT and the risk of ACS became insignificant after adjustment for ox-LDL, we believe that GGT could still be considered as a biomarker of increased cardiovascular risk. Further large population-based cohort studies are needed for confirmation. Lifestyle modifications, such as weight loss and physical activity, can lower the GGT level [29]. Several pharmacological agents, including pentoxifylline, silymarin, and vitamin E, are also available for modification of the GGT level [30, 31]. However, whether such interventions can help to prevent CVD requires further evaluation.

This study has some limitations. First, the case-control design does not permit determination of causality. Further Mendelian randomization studies of genetic variants specifically related to GGT levels may provide strong evidence to assess the causality of GGT on the risk of ACS. Second, we did not have complete data on ultrasound or other imaging techniques for evaluation of fatty liver disease. Therefore, some patients with fatty liver disease might not have been identified in our study, which might bias the results toward exaggeration of the ACS risk estimates for GGT. However, some other studies have shown that serum GGT levels are associated with cardiovascular risk, independent of fatty liver [27, 32]. Third, the levels of ALT and AST are important biomarkers of hepatitis. In our study, the levels of ALT and AST in the ACS group were significantly higher than those in the control group; this is mainly due to cardiomyocyte necrosis caused by acute cardiac ischemia. However, because other types of hepatitis virus antigens were not tested in every participant, we only excluded patients with history of hepatitis or positive detection of serum hepatitis B virus antigen. Some patients with other types of hepatitis may have not been excluded and could cause potential bias. However, except for hepatitis B, other types of hepatitis were not common in our area.

## 5. Conclusions

GGT activity is associated with the risk of ACS in young Chinese patients, independent of conventional cardiovascular risk factors. The link between GGT and the risk of ACS may be dependent on ox-LDL levels, indicating that the prooxidant action is an important pathway for GGT in the development of cardiovascular disease.

## Figures and Tables

**Table 1:** Demographic and clinical characteristics of patients.

	Control group (*n* = 227)	ACS group (*n* = 216)
Age (years)	50.2 ± 10.6	48.6 ± 8.5
CAD family history [*n* (%)]	18 (7.9%)	15 (6.9%)
Current smokers [*n* (%)]	35 (15.4%)	85 (39.4%)^#^
Past smokers [*n* (%)]	8 (3.5%)	10 (4.6%)
Pack years of smoking	6.8 ± 4.5	15.3 ± 8.2^#^
Men [*n* (%)]	110 (50.7%)	168 (77.8%)^#^
Hypertension [*n* (%)]	38 (16.7%)	45 (20.8%)
Systolic blood pressure (mmHg)	123.0 ± 13.9	121.5 ± 12.8
Diastolic blood pressure(mmHg)	73.4 ± 8.7	74.5 ± 8.5
Diabetes mellitus [*n* (%)]	20 (8.4%)	24 (11.1%)
Fasting blood glucose (mg/dL)	92.3 ± 15.8	95.4 ± 16.2
Dyslipidemia [*n* (%)]	58 (25.6%)	72 (33.3%)^∗^
Triglyceride (mg/dL)	150.8 ± 28.0	179.1 ± 28.4^#^
Total cholesterol (mg/dL)	184.2 ± 39.6	188.4 ± 38.4
Low-density lipoprotein cholesterol (mg/dL)	126.2 ± 26.3	130.6 ± 28.5
High-density lipoprotein cholesterol (mg/dL)	43.3 ± 13.8	41.2 ± 13.4
Overweight/obesity [*n* (%)]	63 (27.9%)	72 (33.3%)
Body mass index (kg/m^2^)	23.2 ± 5.6	24.5 ± 6.1
eGFR (ml/min/1.73 m^2^)	115.1 ± 39.7	111.0 ± 35.8
hs-CRP (mg/dL)	2.21 (0.75–7.65)	5.54 (1.14–10.31)
GGT (U/L)	21.5 (14.3–55.3)	36.7 (16.8–78.6)^#^
AST (U/L)	29 (18–47)	135 (37–265)^#^
ALT (U/L)	26 (12–42)	52 (18–124)^#^
TBIL (*μ*mmol/L)	12.8 ± 6.8	13.7 ± 6.5
DBIL (*μ*mmol/L)	3.7 ± 2.5	4.0 ± 2.7
IBIL (*μ*mmol/L)	9.2 ± 5.0	9.7 ± 5.8
Ox-LDL(mg/dL)	1.20 ± 0.83	3.24 ± 1.74^#^
10-year CVD event risk (%)	5.1 ± 3.9	5.6 ± 3.5

Categorical variables were presented as percentages; continuous variables were expressed as median (interquartile range) or mean ± standard deviation. ACS, acute coronary syndrome; ALT, alanine aminotransferase; AST, aspartate aminotransferase; CVD, cardiovascular disease; DBIL, direct bilirubin; eGFR, estimated glomerular filtration rate; GGT, gamma-glutamyltransferase; hs-CRP, high-sensitivity C-reactive protein; IBIL, indirect bilirubin; ox-LDL, oxidized low-density lipoprotein; TBIL, total bilirubin; ^∗^*P* < 0.05, ^#^*P* < 0.01.

**Table 2:** Correlation of gamma-glutamyltransferase and conventional cardiovascular risk factors.

Variables	*r* value	*P* value
Age	0.08	0.76
Sex	−0.15	0.17
Current smoking	0.16	0.13
Pack years of smoking	0.18	0.11
Systolic blood pressure	0.08	0.73
Diastolic blood pressure	0.05	0.85
Fasting blood glucose	0.22	0.02
TC	0.20	0.15
LDL-C	0.14	0.25
HDL-C	−0.17	0.11
Triglyceride	0.23	0.01
Body mass index	0.18	0.04
eGFR	0.15	0.11
Log_e_ hs-CRP	0.19	0.19
Log_e_ AST	0.23	0.04
Log_e_ ALT	0.21	0.07
TBIL	−0.17	0.13
DBIL	−0.09	0.65
IBIL	0.12	0.23
Ox-LDL	0.32	<0.001

eGFR, estimated glomerular filtration rate; HDL-C, high-density lipoprotein cholesterol; hs-CRP, high-sensitivity C-reactive protein; LDL-C, low-density lipoprotein cholesterol; ox-LDL, oxidized low-density lipoprotein; TC, total cholesterol.

**Table 3:** Risk factors for ACS in young patients in multivariate logistic regression analysis.

Risk factors	OR	95% CI	*P* value
Sex (male vs. female)	3.45	1.21–9.84	0.021
Age (per 10 years)	1.29	0.90–1.85	0.167
Pack years of smoking (≥10 vs. <10)	2.79	1.48–5.30	0.002
CAD family history (yes vs. no)	1.08	0.24–4.66	0.921
Overweight/obesity (yes vs. no)	1.17	0.89–1.54	0.261
Diabetes mellitus (yes vs. no)	1.46	0.92–2.32	0.108
Hypertension (yes vs. no)	1.30	0.90–1.88	0.162
Triglyceride (per SD)	1.48	1.03–2.13	0.034
TC (per SD)	1.27	0.87–1.85	0.216
LDL-C (per SD)	1.31	0.95–1.81	0.10
HDL-C (per SD)	0.89	0.30–2.64	0.834
eGFR (per SD)	0.87	0.52–1.46	0.60
Log_e_ hs-CRP (per SD)	2.36	1.18–4.72	0.015
Log_e_ GGT (per SD)	1.53	1.09–2.15	0.014

For logistic regression, smoking history was quantified as pack years and dichotomized at the median value (10.0) of all subjects. CVD: cardiovascular disease; CI, confidence interval; eGFR: estimated glomerular filtration rate; HDL-C: high-density lipoprotein cholesterol; hs-CRP: high-sensitivity C-reactive protein; LDL-C: low-density lipoprotein cholesterol; OR: odds ratio; TC: total cholesterol.

## Data Availability

The research data used to support the findings of this study are currently under embargo. Requests for data, 12 months after publication of this article, will be considered by the corresponding author, Dr. Yunzhao Hu, Department of Cardiology, Shunde Hospital, Southern Medical University, Penglai Road, Daliang Town, Shunde District, Foshan 528300, China. E-mail: huyunzhao4406@163.com.
